# Expression analysis of vitellogenins in the workers of the red imported fire ant (*Solenopsis invicta*)

**DOI:** 10.7717/peerj.4875

**Published:** 2018-05-28

**Authors:** Chloe Hawkings, Cecilia Tamborindeguy

**Affiliations:** Department of Entomology, Texas A&M University, College Station, TX, USA

**Keywords:** Red imported fire ant, Social insect, Vitellogenin, Worker caste, Juvenile hormone, Task allocation, Ants, Gene expression

## Abstract

Vitellogenin has been proposed to regulate division of labor and social organization in social insects. The red imported fire ant (*Solenopsis invicta*) harbors four distinct, adjacent vitellogenin genes (Vg1, Vg2, Vg3, and Vg4). Contrary to honey bees that have a single Vg ortholog as well as potentially fertile nurses, and to other ant species that lay trophic eggs, *S. invicta* workers completely lack ovaries or the ability to lay eggs. This provides a unique model to investigate whether Vg duplication in *S. invicta* was followed by subfunctionalization to acquire non-reproductive functions and whether Vg was co-opted to regulate behavior within the worker caste. To investigate these questions, we compared the expression patterns of *S. invicta* Vg genes among workers from different morphological subcastes or performing different tasks. RT-qPCRs revealed higher relative expression of Vg1 in major workers compared to both medium and minor workers, and of Vg2 in major workers when compared to minor workers. Relative expression of Vg1 was also higher in carbohydrate foragers when compared to nurses and protein foragers. By contrast, the level of expression of Vg2, Vg3, and Vg4 were not significantly different among the workers performing the specific tasks. Additionally, we analyzed the relationship between the expression of the Vg genes and S-hydroprene, a juvenile hormone analog. No changes in Vg expression were recorded in workers 12 h after application of the analog. Our results suggest that in *S. invicta* the Vg gene underwent subfunctionalization after duplication to new functions based on the expression bias observed in these data. This may suggest an alternative and still unknown function for Vg in the workers that needs to be investigated further.

## Introduction

Division of labor and foraging specialization are a key characteristic of the eusocial insect colony structure. Many factors can influence division of labor in insect societies, such as morphology, genetic variation, developmental and nutritional factors, experience, and age (Kohlmeier et al., 2017; Libbrecht et al., 2013; Page & Robinson, 1991; Robinson, 1987; Toth & Robinson, 2007). In social insect colonies, vitellogenin (Vg) may control the division of labor, social behavior, and colony function (Beshers & Fewell, 2001; Guidugli et al., 2005; Nelson et al., 2007; Robinson, Fernald & Clayton, 2008; Smith et al., 2008). Indeed, in the honey bee worker caste Vg is involved in royal jelly production (Amdam et al., 2003), age polyethism regulation (Marco Antonio et al., 2008), antioxidant and immunity regulation, and insulin/insulin-like signaling which controls growth, aging, and reproduction (Amdam et al., 2006; Keller & Jemielity, 2006). This relationship between Vg and task has also been recognized across species of ants (Corona et al., 2013; Graff et al., 2007; Hartmann & Heinze, 2003; Martinez & Wheeler, 1991).

Insects encode a variable number of Vg genes depending on the species. In social insects, difference in Vg expression among castes could play a key role in social organization as proposed by the ovarian ground plan and genetic “toolkit” hypotheses (Toth & Robinson, 2007; West-Eberhard, 1987). Similarly, Vg duplication and subsequent subfunctionalization could be linked with Vg’s role in social organization. Differences in Vg and Vg-like gene expression between queens and workers were identified in several ant species (Feldmeyer, Elsner & Foitzik, 2014; Morandin et al., 2014; Oxley et al., 2014) and differences in Vg expression between workers performing different tasks also exist (Corona et al., 2013). Two Vg subfamilies were identified in the formicoid clade, subfamily A are Vg genes expressed at higher level in queens than in workers, and subfamily B are Vg genes preferentially expressed in workers (Corona et al., 2013). The Red imported fire ant (*Solenopsis invicta* Buren; Hymenoptera: Formicidae) genome harbors four adjacent Vg genes (Vg1, Vg2, Vg3, and Vg4) (Wurm et al., 2011), Vg1 and Vg4 clustering in the B subfamily and Vg2 and Vg3 clustering in the A subfamily (Corona et al., 2013). Previously, it was established that Vg1 and Vg4 transcripts are highly expressed in workers, while Vg2 and Vg3 are expressed at higher level in queens (Wurm et al., 2011), and SDS–PAGE analyses determined that Vg proteins are present in the hemolymph of reproductive queens, virgin alate queens, and workers (Lewis et al., 2001). However, the expression of these genes among task-allocated workers or different subcastes has not been studied.

As for other eusocial insects, *S. invicta* colonies are composed by individuals belonging either to the reproductive caste (queens) or the worker (sterile) caste. The workers are central to colony maintenance and growth, their labor in turn is a central resource through which ants acquire a territory, defend it, and search for food sources to distribute to the entire colony. The workers show little variation in anatomical features, but are highly variable in body length ranging from 2 to 6 mm and are further categorized into subcastes according to their head width and size; these subcastes are known as major, medium, and minor workers (Tschinkel, 2006). The size of the workers depends upon the age of the colony, founding status of a colony, and it may also vary with food availability (Tschinkel, 2006). When colonies are newly established and small, workers are monomorphic and consist exclusively of minors, but as the colonies increase in size and age, approximately one month after colony establishment, the variation in size of the workers becomes increasingly noticeable as does the variation in tasks being performed. As colonies grow, the worker population shifts to an even larger fraction of large workers, suggesting that such shifts in the labor economy could be important to colony growth and reproduction (Tschinkel, 2006). While the molecular basis of task allocation in fire ants has not been elucidated, a correlation between the worker size and the labor performed was described, and in general younger and smaller ants concentrate more on brood care while older and larger ants tend toward foraging (Wilson, 1978). Medium sized workers are the generalists, performing nearly all colony tasks. They are the most versatile and can engage in recruitment to food sources, larval grooming, and larval feeding. They display considerable variation in the frequency in which they feed larvae before switching to other tasks, which suggests flexibility in tasks (Cassill & Tschinkel, 1999). The major workers play a small role in larval care, while the minor workers play a larger role. In addition to nurses and foragers, a third group of workers of heterogeneous age, size, and behavior exists, these ants are denominated as reserves, and are a transitional group from nurses to foragers (Mirenda & Vinson, 1981). This group does not conduct any specific task, but may nurse, forage, store liquid food, and/or relay food between nurses and foragers (Mirenda & Vinson, 1981; Tschinkel, 2006). Depending on the size of the colony, reserves might represent up to 30% of the workers (Tschinkel, 2006; Wilson, 1978). The amount of activity undertaken by each individual worker is different. On average, workers performing specific tasks were inactive 20 min of 30 min observation intervals (Mirenda & Vinson, 1981). Studies analyzing crop content of workers of a monogyne colony, a colony with only one mated queen, determined that workers of any size could switch tasks to food storage and source collection from larval care (Cassill & Tschinkel, 1999). Differences in Vg expression between queens and workers exist in *S. invicta*, however, it is unknown whether there are there differences in Vg expression among the morphological worker subcastes, and/or among workers performing different tasks.

In many insects juvenile hormone (JH) regulates Vg expression (Tufail et al., 2014). In social Hymenoptera, JH is involved in reproductive division of labor as well as in worker age-related division of labor, and differences in the role of JH between primitive and advanced eusocial species exist. For instance, in *Bombus terrestris*, a primitive eusocial insect in which JH retains its gonadotropic role, no changes in worker Vg expression were measured following JH application (Amsalem et al., 2014). On the other hand, an inverse relationship between JH and Vg exists in honey bee workers, and this relationship is involved in the behavioral switch as workers age: JH increases as a worker transitions from nursing to foraging while Vg protein level decreases. Furthermore, Vg is involved in the control of JH synthesis and the feedback loop between JH and Vg regulates the onset of the foraging behavior (Amdam et al., 2003, 2005; Amdam & Omholt, 2002; Guidugli et al., 2005; Marco Antonio et al., 2008). Overall, few studies have assessed the role of JH in Vg regulation in workers. For example, topical application of JH to non-reproductive *Ectatomma tuberculatum* workers resulted in the downregulation of Vg protein synthesis and reduced Vg titers in the hemolymph (Azevedo et al., 2016). While JH has retained its gonadotropic role in the queen (Chen et al., 2004), it is widely unknown if the interplay of JH and Vg is important in regulation of tasks in workers of *S. invicta*, or whether Vg expression is regulated by JH in workers.

*S. invicta* is an ideal species to evaluate the role of Vg in task allocation because the workers are sterile, and therefore the role of these genes in reproduction and task allocation can be decoupled. The goals of this study were: (1) to investigate the expression of the four Vg transcripts in the three worker subcastes (minor, medium, and major) of *S. invicta*; (2) to investigate the expression of the four Vg transcripts in workers performing different tasks (nursing, foraging carbohydrates, and foraging proteins); and (3) to identify the potential role of JH in the expression of each of the four Vgs using topical applications of a JH analog.

## Materials and Methods

### Insect colonies

Polygyne colonies of *S. invicta* were collected in Brazos County, TX from May to July 2015 and maintained in the laboratory in plastic trays (27 × 40 × 9 cm) with the walls of the containers covered with Fluon (Insect-a-slip; BioQuip products, Compton, CA, USA) in the Department of Entomology at Texas A&M University, College Station, TX, USA. The colonies were maintained at 27 ± 2 °C in a 12:12 h light–dark photoperiod. Ant colonies were provided with a 14 cm diameter petri dish half filled with damp Castone (Dentsply International Inc., York, PA, USA) as a nest area. Ant colonies were fed daily with a 20% honey water solution and crickets, *Acheta domestica*. Water was given ad libitum. Colonies contained mated queens, alate queens, males, brood (eggs, larvae, and pupae), and a polymorphic worker caste.

### Classification and selection of worker ants

**Figure 1:**
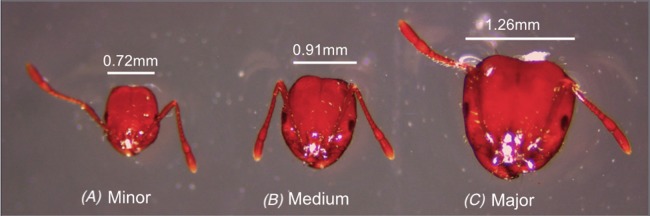
Comparison of the head width among individuals from the worker caste of a single colony (A) Minor, (B) Medium, (C) Major. Photography credit: Chloe Hawkings.

For subcaste analyses, ants were classified into majors, mediums or minors according to their head width, as previously described by Wilson (1978). Minor workers had a head width smaller than 0.72 mm; medium workers’ head width was between 0.73 and 0.92 mm; and major workers had a head width larger than 0.93 mm (Fig. 1).

For task-allocated ant analyses, medium workers were collected while conducting a specific task: protein foraging, carbohydrate foraging, or nursing. Ants interacting with brood were considered as nurses. Foragers were determined as ants interacting directly with the specific food type. Ants were collected 30 min after the food source was renewed for the day. So, if a worker was found actively collecting food from the protein tray at this time it was classified as a protein forager, if a worker was found on the carbohydrate source it was classified as a carbohydrate forager, and if the worker was in the nest interacting with the brood at the time of food source introduction it was classified as a nurse. Food sources were replaced at approximately 9:00 AM, so ants for all experiments were collected between 9:30 and 10:00 AM.

Five different colonies were used for the morphological caste and task allocation Vg transcript expression assays (five biological replicates each). All replicates were collected within one month; this was done to prevent variations caused by natural circadian rhythms. For each experimental replicate, 10 ants were collected from the same colony. For the task allocation assay, medium workers were selected because of their versatility in the tasks they perform. Pools of 10 workers were flash-frozen in liquid nitrogen upon collection and kept at −80 °C until further use.

### Selection of specific primer for each Vg

Primers for each Vg gene were designed using the UGENE software (Okonechnikov et al., 2012). The four Vg transcript sequences were aligned using the UGENE alignment tool and specific regions of each Vg were identified. Primers were designed manually to amplify the specific regions. To further verify the accuracy of the qPCR primers (Table 1), DNA was extracted from whole body worker ants using the E.Z.N.A. Insect DNA kit (OMEGA Bio-Tek, Norcross, GA, USA). PCR was conducted using the following optimized temperature parameters: 94 °C for 2 min, then 35 cycles at 94 °C for 15 s, 60 °C for 15 s, and 68 °C for 30 s, followed by a final elongation step of 5 min at 68 °C. The reaction was conducted in a 50 μL volume containing 1× GoTaq Green Master Mix (Promega, Madison, WI, USA) and 0.4 nM of each Vg primer. The PCR products were separated in a 2% agarose gel, and then purified using the PureLink PCR purification kit (Invitrogen, Carlsbad, CA, USA), following the manufacturer’s protocol. The amplicons were cloned into the pGEM^®^-T easy vector (Promega, Madison, WI, USA). Plasmids were introduced into One Shot^®^ TOP10 Chemically Competent *E. coli* (Invitrogen) and purification was conducted using the PureLink Quick Plasmid MiniPrep Kit (Invitrogen, Carlsbad, CA, USA) following the manufacturer’s protocol. Five bacterial colonies were selected from each plate and sent to Eton Biosciences (San Diego, CA, USA) for sequencing. Sequences were analyzed using BLAST which confirmed the correct vitellogenin for each primer set.

**Table 1:** Primers used for gene expression analysis.

Name	Sequence
SiVg1_F	5′-CTTACCATTCTGGCATCACC-3′
SiVg1_R	5′-GGGCAATAACGGACTCTCTG-3′
SiVg2_F	5′-CATGTGGTTCCCTGTCACC-3′
SiVg2_R	5′-GACTCGTCGCTAGGAACCTG-3′
SiVg3_F	5′-TATCGAACGGTCCGTATTCCA-3′
SiVg3_R	5′-TCGTGGATAATTCCGAAACA-3′
SiVg4_F	5′-AGTCGAGCCCCCAAAAGC-3′
SiVg4_R	5′-GATGAGAGCGGGTCCAGTT-3′

### Gene expression analysis

Pools of 10 whole body insects were used for gene expression analyses. Pools were used to normalize the natural variation in gene expression in the colony, in addition they yielded a sufficient quantity of RNA for each sample. Insects in each biological replicate were ground in liquid nitrogen with a pestle and mortar into a fine powder. Total RNA extractions were performed using the Trizol reagent (Invitrogen, Carlsbad, CA, USA) following the manufacturer’s instructions. RNA purification was completed using the Micro prep plus clean up kit (Zymo Research, Irvine, CA, USA) for sample clean-up. The RNA was resuspended in 20 μL of nuclease-free water. Genomic DNA was eliminated with the Turbo DNAse kit (Ambion, Waltham, MA, USA) following the manufacturer’s instructions. Total RNA quantity and purity was assessed using an Infinite^®^ 200 PRO NanoQuant (Tecan, Männedorf, Switzerland) and RNA integrity was visualized by electrophoresis using a 2% agarose gel stained with ethidium bromide.

For expression analyses, RT-qPCR reactions were performed using the SensiFAST SYBR^®^ Hi-rox one step kit (Bioline, Taunton, MA, USA) according to the manufacturer’s instructions. Each reaction contained 50 ng of RNA, 250 nM of forward and reverse primer; and 1× of SYBR Green Master Mix; the volume was adjusted with nuclease-free water to 10 μL. The thermocycler program was 45 °C for 10 min followed by 95 °C for 2 min and 40 cycles at 95 °C for 5 s and 60 °C for 30 s. Real-time PCR assays were performed using an Applied Biosystems ABI 7300 real-time PCR Thermocycler (Applied Biosystems, Foster City, CA, USA) according to manufacturer’s recommendations. Reactions for all samples were performed in duplicates with negative controls for each reaction. The threshold cycle (Ct) values and the efficiency of each primer set for RT-qPCR were determined using LinRegPCR software (Ramakers et al., 2003) and primer specificity was monitored with the melt curve analysis using the Sequence detection system version 1.4.0.27 (Applied Biosystems, Foster City, CA, USA). The relative expression of each vitellogenin gene was estimated with the delta delta CT method (Schmittgen & Livak, 2008) by normalizing the levels of each Vg transcript to the internal control. Six putative housekeeping genes (ribosomal protein 9 (*RP9*), ribosomal protein L18 (*RP18*), translation elongation factor 1 (*ef1-beta*), actin, glyceraldehyde-3-phosphate dehydrogenase (*GAPDH*), and TATA box binding protein (*tbp*)) (Cheng et al., 2013; Wurm et al., 2011) were tested among subcastes. Only *RP18* displayed low variation among groups, and was thus used to normalize Vg expression values. For each Vg gene, the samples were calibrated using the minor subcaste or the carbohydrate forager relative expression in the Vg expression studies.

### Juvenile hormone analog study

Major workers (1.26 mm head width) were collected and were treated topically on the abdomen with 1 μL of S-hydroprene (Sigma-Aldrich, St. Louis, MO, USA) (25 ng/μL dissolved in 80% acetone and 20% ethanol), a JH-mimic, or with 1 μL of a solvent (80% acetone and 20% ethanol; solvent control) once during the assay. This S-hydroprene dose was similar to that used previously to produce realistic changes in JH titers for other social insect workers (Cahan, Graves & Brent, 2011; Pamminger et al., 2016; Shorter & Tibbetts, 2009). Fresh solutions of S-hydroprene were prepared for each biological replicate. Another group of ants were similarly manipulated but without any topical application (untreated control). After topical application of the S-hydroprene or the control solutions, treated and untreated ants were caged according to their treatment in a container within the original colony. Six different colonies were used as biological replicates for the JH assay (six replicates). After 12 h following treatment, 10 worker ants from each treatment were removed, pooled, flash frozen and kept at −80 °C until further analyses. RNA purification and expression analyses by RT-qPCR of Vg1, Vg2, Vg3, and Vg4 were performed as previously described. All qPCR reactions were conducted in duplicates and assays were performed in six biological replicates. For the expression analysis, the untreated control was used to calibrate the Vg relative expression. As a positive control to validate the application of the JH analog to *S. invicta*, virgin alate queens were tested following the protocol by Vargo and Laurel (Vargo & Laurel, 1994) since JH application to alate virgin queens causes them to dealate. Twelve hours following the topical application virgin queens were checked for dealation.

### Statistical analysis

All data are reported as means; error bars represent standard error of the mean. Statistical analyses were performed with the one-way ANOVA test and Tukey–Kramer post hoc using the JMP Version 13 (SAS Institute Inc., Cary, NC, USA, 1989–2017); estimated *p*-values were considered significant below the 0.05 threshold.

### Protein domain analysis of vitellogenin

Vg protein sequences were downloaded from the NCBI database (ID numbers LOC105205865, LOC105205782, LOC105205783). LOC105205865 appears to encode both Vg1 and Vg4 proteins (see “results” section). Protein domains were identified by searching the NCBI Conserved Domain Database (CDD) (Marchler-Bauer et al., 2014). Signal peptides were identified using SignalP (Petersen et al., 2011). Protein structure was visualized in IBS (Liu et al., 2015).

## Results

### Protein domain analysis of the vitellogenin proteins

**Figure 2:**
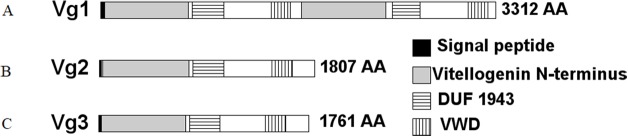
Structural domains identified in the *S. invicta* predicted Vg proteins, (A) Vg1, (B) Vg2, (C) Vg3. Each protein encoded a putative cleaved signal peptide (black). These proteins possess the LPD-N (gray), DUF1943 (horizontal stripes), and VWD (vertical stripes) domains commonly found in Vg proteins. While Vg2 and Vg3 possessed one of each domain, Vg1 possessed two. Therefore, Vg1 might encode Vg1 and Vg4.

Three sequences were identified in NCBI as Vg genes, LOC105205865, LOC105205782, and LOC105205783. These genes are annotated as Vitellogenin-1, Vitellogenin-2, and Vitellogenin-3, respectively, and are contiguous in *S. invicta* genome in the location NW_011804688.1. The encoded proteins were 3,312, 1,807, and 1,761 amino acids (AAs) in length, respectively. Further analyses of these proteins revealed that Vg1 encoded a putative signal peptide predicted to be cleaved at position 29, two Vitellogenin N-terminus (lipoprotein amino terminal region) domains (AAs 34–736 and 1684–2,389), two domains of unknown function (DUF1943) (AAs 770–1,032 and 2,445–2,677), and two von Willebrand factor type D (VWD) domains (AAs 1,428–1,591 and 3,079–3,247) (Fig. 2). However, Vg2 and Vg3 had each a predicted signal peptide cleaved after AA 16, one Vitellogenin N-terminus domain (AAs 26–751 and 24–730, respectively), one DUF1943 (AAs 784–1,043 and 764–1,016, respectively), and one VWD domain (AAs 1,444–1,615 and 1,397–1,570, respectively). Based on these results, we determined that both Vg1 and Vg4 genes were merged under the same ID number and that the Vg1 and Vg4 proteins have been merged (accession number XP_011173700.1). Indeed, inspection of the LOC105205865 sequence revealed a 350-base pair gap approximately at position 2,330,945 in the NW_011804688.1 location, therefore we concluded that Vg1 corresponds to AA 1–1,662, and Vg4 to AA 1,663–3,312 approximately.

### Vitellogenin expression in morphological subcastes

**Figure 3:**
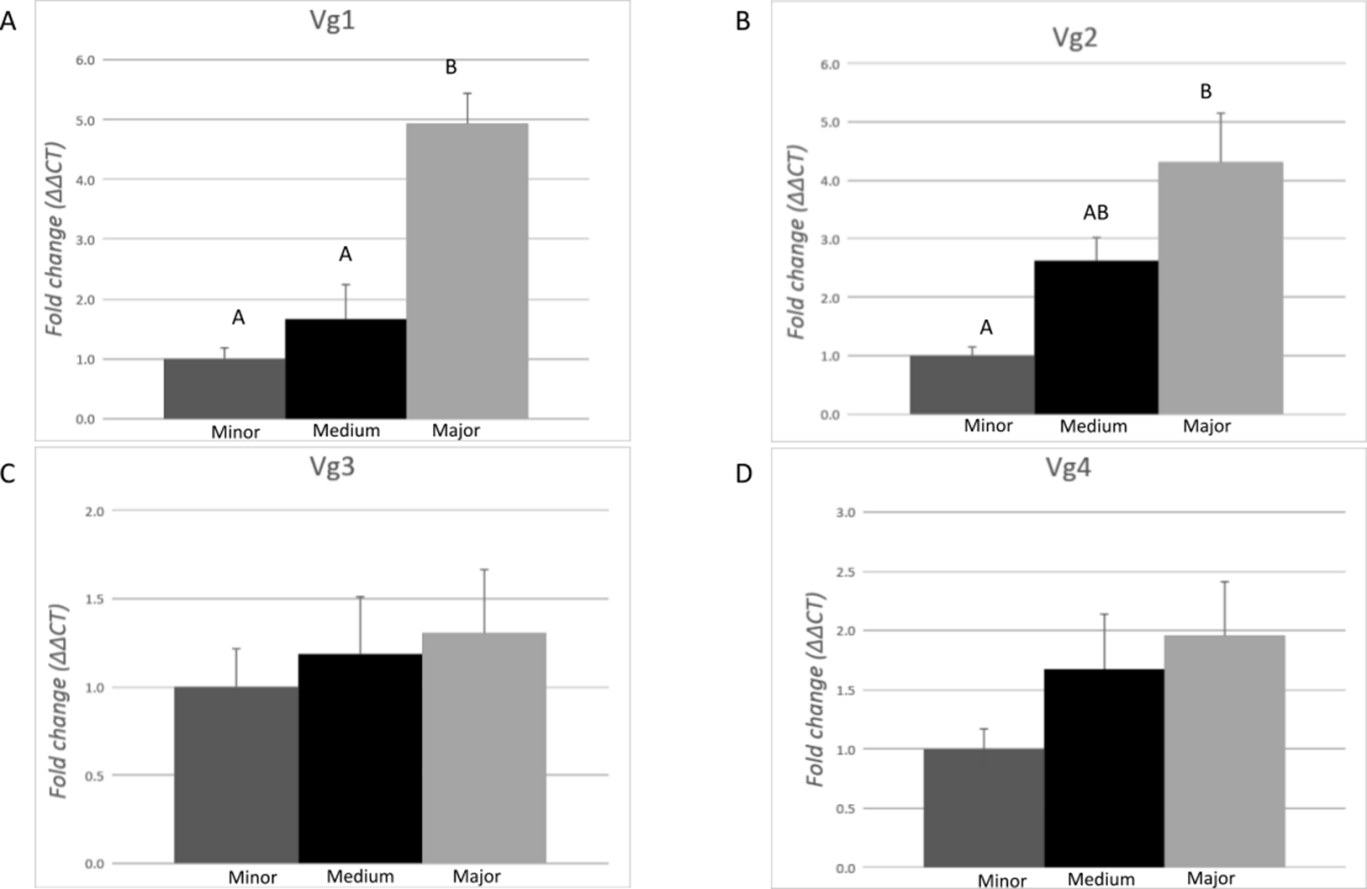
Expression analysis of the vitellogenin transcripts among the worker morphological subcastes. Each bar represents the mean ± SEM (*n* = 5). All workers were carbohydrate foragers. (A) Vg1 transcript expression. (B) Vg2 transcript expression. (C) Vg3 transcript expression. (D) Vg4 transcript expression. Vg mRNA expression level was normalized relative to RP18 mRNA expression level. Statistical relationships between groups were assessed using one-way ANOVA with Tukey–Kramer post hoc test (*p* < 0.05), where different letters indicate statistical differences among the subcastes.

The expression profile of each Vg transcript (Vg1, Vg2, Vg3, and Vg4) was evaluated in pools of 10 ants from each morphological subcaste of the worker caste conducting the task of carbohydrate foraging by RT-qPCR (Fig. 3). Differences of expression among the subcastes were identified for Vg1 (*F* = 20.93, df = 2.00, *p* = 0.0001) and Vg2 (*F* = 10.03, df = 2.00, *p* = 0.0027). On average, expression of Vg1 was 4.6- and 2.9-fold higher in major workers relative to minor and medium workers, respectively. There were no significant differences (*p* > 0.05) in Vg1 expression between minor and medium ants. On average, Vg2 was 4.8-fold higher in major workers than in minor workers. No differences in the expression of Vg2 were measured between medium and minor workers or between medium and major workers. No differences in the expression of Vg3 and Vg4 were measured among the morphological worker subcastes.

### Vitellogenin expression in task allocated medium workers

**Figure 4:**
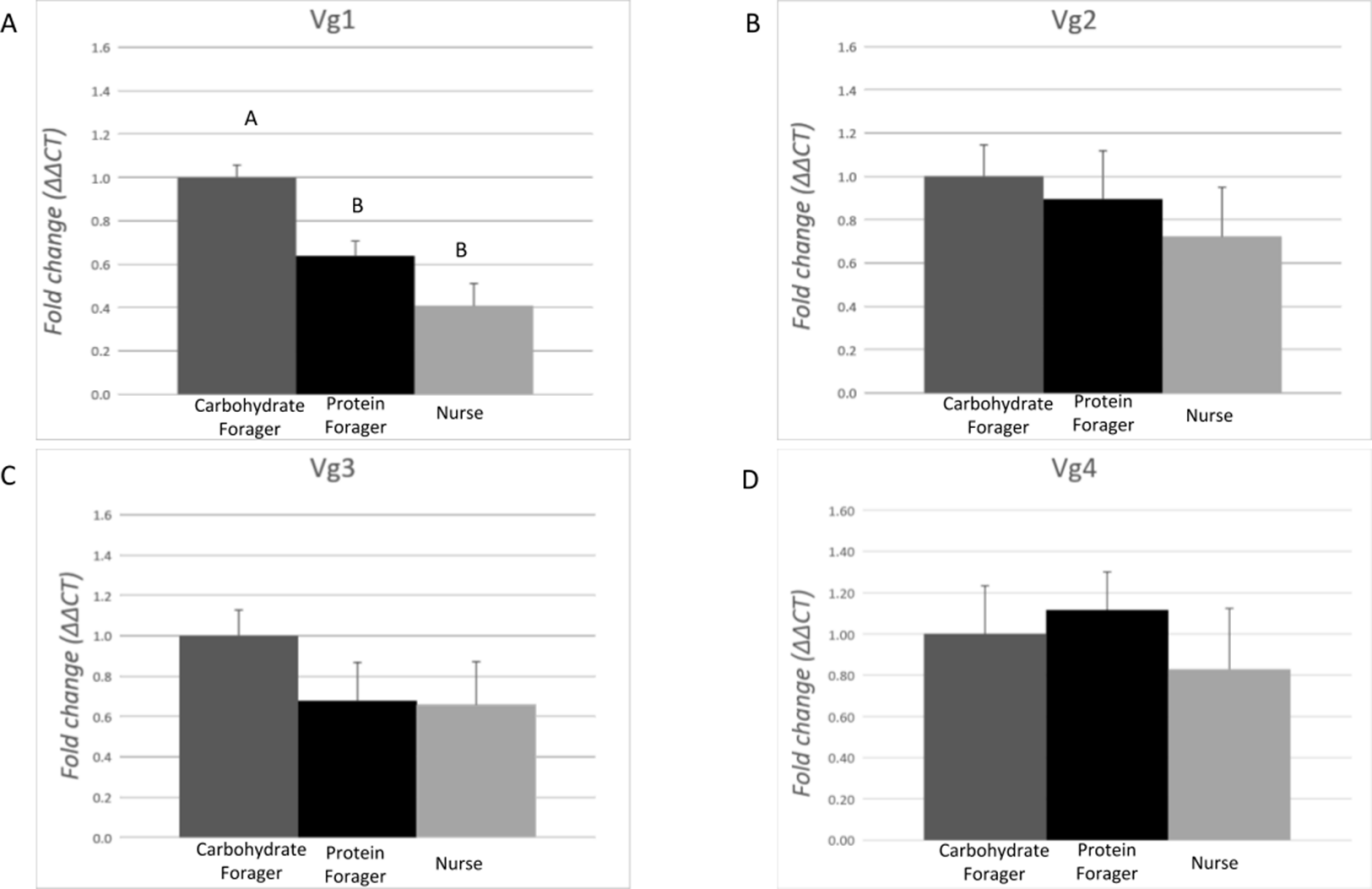
Expression analysis of the vitellogenin transcripts among medium workers performing different tasks. Each bar represents the mean ± SEM (*n* = 5). (A) Vg1 transcript expression. (B) Vg2 transcript expression. (C) Vg3 transcript expression. (D) Vg4 transcript expression. Vg mRNA expression level was normalized relative to RP18 mRNA expression level. Statistical relationships between groups were assessed using one-way ANOVA with Tukey–Kramer post hoc test (*p* < 0.05), where different letters indicate statistical differences among the different task-allocated insects.

The expression level of each Vg transcript was evaluated in task-allocated medium workers performing specific tasks: nurses (non-foraging), carbohydrate foragers and protein foragers (Fig. 4). Differences of expression among the ants performing specific tasks were identified for Vg1 (*F* = 14.90, df = 2.00, *p* = 0.0006). On average, expression of Vg1 was 2.4- and 1.5-fold higher in carbohydrate foragers relative to nurses and protein foragers, respectively, while no differences of Vg1 expression were measured between protein foragers and nurses. There were no differences in Vg2, Vg3, or Vg4 relative expression among the task-allocated medium workers.

### Vitellogenin expression in major workers after application with S-hydroprene

**Figure 5:**
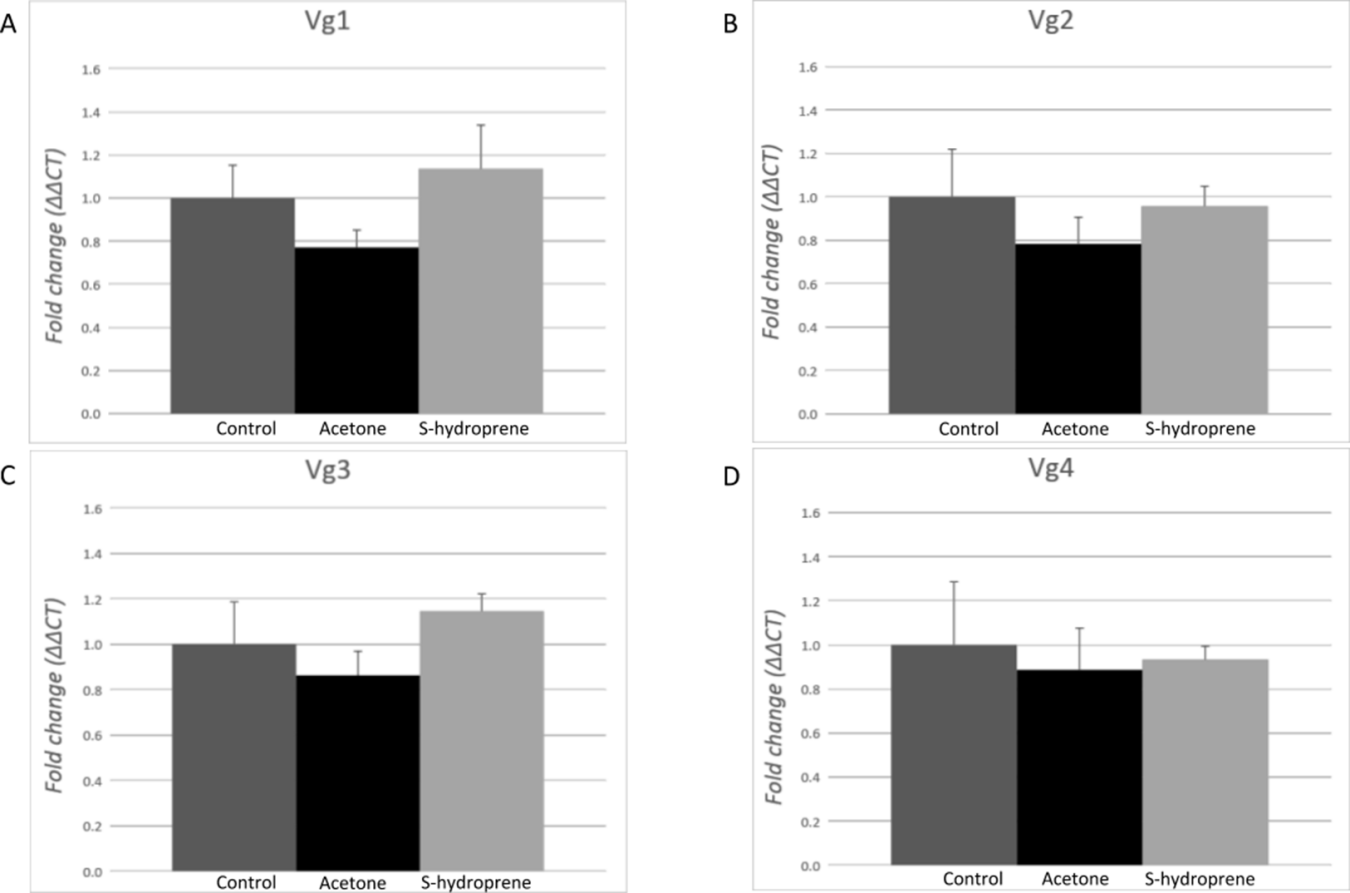
Expression analysis of the vitellogenin transcripts among major workers 12 h after topical application of S-hydroprene. Each bar represents the mean ± SEM (*n* = 6). (A) Vg1 transcript expression. (B) Vg2 transcript expression. (C) Vg3 transcript expression. (D) Vg4 transcript expression. Vg mRNA expression level was normalized relative to RP18 mRNA expression level. Statistical relationships between groups were assessed using one-way ANOVA; no significant differences were found for any of the Vg transcripts (*p* > 0.05).

No changes in the expression of any of the Vg transcripts were measured 12 h after the topical application of S-hydroprene in task-allocated (carbohydrate foragers) major workers (Fig. 5). No significant differences were found between the non-treatment control and acetone, and no significant differences were found between S-hydroprene and acetone or S-hydroprene and the control treatment (*p* > 0.05).

**Figure 6:**
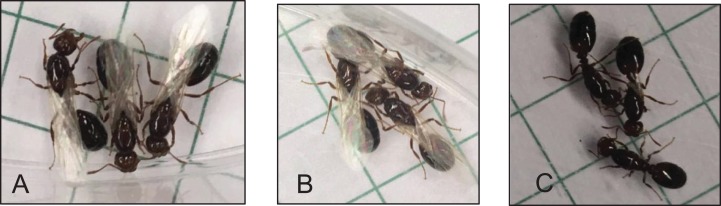
Evaluation of S-hydroprene application and effect in alate virgin queens 12 h after treatment. (A) Untreated alate queens, 0% of the tested virgins dealated. (B) Alate queen treated with acetone, 0% of the tested virgin dealated. (C) Alate queens treated with S-hydroprene, 100% of the tested virgins dealated. Photography credit: Chloe Hawkings.

Topical application of S-hydroprene resulted in 100% dealation of virgin queens, while no virgin treated with the acetone control solution or in the untreated control dealated (Fig. 6).

## Discussion

The present studies were undertaken to characterize the expression of the four Vg genes in *S. invicta* workers as a first step to investigating the functional role they may play in the worker caste. Changes in Vg sequence following duplication could be related to neofunctionalization in social insects. Because sequence evolution is faster in genes with a caste-biased expression in *S. invicta* than in genes with unbiased expression (Hunt et al., 2011), it was important to evaluate if the *S. invicta* Vg genes encoded the typical Vg domains. The four predicted *S. invicta* Vg genes encoded the typical Vg domains, lipoprotein N-terminal domain, DUF1943, and VWD. The roles of these domains have not been identified so far, however, DUF1943 and VWD can recognize lipopolysaccharides and lipoteichoic acid from bacteria and may be involved in pattern recognition and Vg’s role in immunity (Sun et al., 2013), while the lipoprotein N-terminal domain is involved in the interaction with the Vg receptor (Li, Sadasivam & Ding, 2003; Roth et al., 2013). Since *S. invicta* workers do not have ovaries and lack the ability to lay either reproductive or trophic eggs (Khila & Abouheif, 2008), the role of Vg proteins in the worker caste remains unknown.

In the present study, differential expression of Vg genes among workers of different sizes (subcaste) and workers performing specific tasks were identified. Vg1 was up-regulated in major workers compared to medium and minor workers, while Vg2 was up-regulated in major workers compared to minor workers. Vg1 was also up-regulated in carbohydrate foragers when compared with nurses and protein foragers. These results suggest that Vg1 after gene duplication and subfunctionalization could have acquired a subcaste- and task-related expression in workers. Our studies also indicated that Vg2 had a subcaste-related expression profile but no differential expression among task-allocated workers.

Two points need to be highlighted. First, Vg is expressed in the fat body (Arrese & Soulages, 2010; Corona et al., 2007), therefore the higher expression in major workers could be related to relative different amounts of fat body among the subcastes. However, in that scenario all Vgs should be expressed at a higher level in majors than in minors, which was not the case. Therefore, the up-regulation of Vg1 and Vg2 in major workers compared to smaller ants could be associated with functional differentiation in the fat body as reported for *Monomorium pharaonic* (Jensen & Borgesen, 2000). Second, based on the phylogenetic relationship of *S. invicta* Vg proteins and on previous analyses of Vg expression in fire ant queens and workers (Wurm et al., 2011), similar expression profiles might have been expected for Vg1 and Vg4 (subfamily Vg B) and for Vg2 and Vg3 (subfamily Vg A). However, the expression pattern in workers was different between the two most similar (most recent duplications) genes.

Differential expression of Vg genes between queens and workers were reported (Wurm et al., 2011), with Vg2 and Vg3 being up-regulated in queens compared to workers, while Vg1 and Vg4 are up-regulated in workers compared to queens, which is consistent with the loss of reproductive constraints and evolution of new functions. Vg3 is shown to be consistently similar throughout subcastes and tasks indicating that this gene could perhaps have a preferential functional role in ovarian activity. Previous studies in *Pogonomyrmex* sp. indicated that Vg expression was differential between foragers and nurses (Corona et al., 2013; Keller & Jemielity, 2006). In *Temnothorax longispinosus* Vg2 and Vg3 are up-regulated in queens compared to foragers, Vg1 is up-regulated in foragers and infertile workers, while foragers have reduced expression of Vg2, Vg3, and Vg6 (Feldmeyer, Elsner & Foitzik, 2014). These results are inconsistent with our study as protein forager expression profiles were shown to be more closely related to nurses than to carbohydrate foragers. A potential explanation for this would be that *Pogonomyrmex* sp. workers can produce trophic eggs which are thought to be the main method of nutrient distribution because trophallaxis has not been observed in this species (Corona et al., 2013). Another recent study in *E. tuberculatum* showed that nurses have significantly increased ovarian activity compared with foragers, suggesting that trophic eggs are produced by the nurses which could ultimately result in the differential expression of Vg in that species (Azevedo et al., 2016). This pattern of nutrient sharing differs from *S. invicta* where the solid protein food sources must be provided to the fourth instar larvae for digestion before redistribution to the remainder of the colony.

Juvenile hormone appears to have maintained its gonadotropic role in primitive eusocial wasps and fire ants but not in other advanced eusocial insects and some other ant species (Bloch, Hefetz & Hartfelder, 2000; Robinson & Vargo, 1997). In adult *A. mellifera* workers, an increase in JH titer in the hemolymph is related to lower Vg titers (Edwards, 1975; Excels, 1974) and topical application of JH inhibits Vg expression (Corona et al., 2007). Our data indicate that *S. invicta* does not follow this pattern of an inverse relationship observed in the honey bee and other ant species (Azevedo et al., 2016; Brent et al., 2006). The JH analog, S-hydroprene, had no effect on the expression of the four Vgs 12 h after topical application on the abdomen of workers. Previously, it was reported that topical application of a JH analog to virgin queens resulted in queen dealation coupled with some degree of ovary development 8 and 12 h after JH analog application to queens (Vargo & Laurel, 1994). It was suggested that in *S. invicta* queens, Vg is constitutive, while yolk formation is regulated through the level of Vg uptake into the oocyte, rather than at the level of Vg synthesis by the fat body (Chen et al., 2004). Also, topical application of S-hydroprene to queens resulted in dealation and down-regulation of a hexamerin-like gene 12 h after treatment (Calkins et al., 2018). Similar dealation results were obtained when we performed the applications of S-hydroprene to queens, which validated our method of JH analog application for the workers. However, visible physiological changes are undetectable in workers because of their lack of wings and ovaries. Our study could also suggest that increased JH may be involved with uptake of the protein rather than increased transcript, consistent with previous studies on the queen in *S. invicta* (Brent & Vargo, 2003; Lewis et al., 2001), which would result in no change at the transcript level. This result could be indicative of the functional component of the duplicated Vg genes. *Solenopsis invicta* have four Vg genes while *A. mellifera* have only one, and the inverse relationship of Vg and JH may be correlated with reproductive capabilities. Our findings open a new avenue to test whether JH has a different regulatory pathway, and its potential influence on age polyethism in *S. invicta.* This study was aimed at evaluating the expression of Vg genes in the whole body of *S. invicta* task-allocated and different subcaste workers. Future studies should analyze the expression of each Vg in specific body regions (Seehuus et al., 2007) of queens and workers and should also explore the potential relationship of JH and Vg in specific body regions or tissues.

## Conclusions

In conclusion, the results of this study suggest that Vg1 is correlated to both subcaste size and task allocation, suggesting that it could have been co-opted to regulate behavior. Vg2 is correlated with subcaste size, potentially suggesting a size-biased expression in the workers, in particular in the major workers, which have a higher expression than smaller workers. Vg3 and Vg4 showed no significant differences in expression among subcaste sizes or task allocation. Vg1 and Vg2 expression pattern among subcastes could be consistent with the relative amount of fat body present in the ants. However, this was not the case for Vg3 and Vg4. This result likely reflects the existence of specific regulation of these genes. Furthermore, it is possible that future analyses performed using age-specific workers or increasing the number of replicates analyzed might uncover more subtle differences in Vg gene expression among subcaste or task-allocated *S. invicta* workers. While expression at a whole body level may not be significantly different, further exploration of Vg expression in specific tissues may reveal subcaste- or task-associated changes in workers. Application of a JH analog had no significant effect on the expression of any of the four Vg genes in workers 12 h after topical application. Overall, these results might support the co-option of reproductive pathways to regulate the behavior of the sterile worker caste, however the role of Vg in the *S. invicta* workers still needs to be elucidated.

## Supplemental Information

10.7717/peerj.4875/supp-1 Supplemental Information 1 Raw data for Vg expression in different fire ant subcastes. Each page contain the raw and analyzed qPCR data for each Vg gene in minor, medium and major carbohydrate fire ant foragers.

10.7717/peerj.4875/supp-2 Supplemental Information 2 Raw data for Vg expression in task allocated ants. Each page contain the raw and analyzed qPCR data for each Vg gene in different task allocated fire ant workers.

10.7717/peerj.4875/supp-3 Supplemental Information 3 Raw data for Vg expression following JH analog application. Raw and analyzed qPCR data for each Vg gene in control, acetone treated and JH analog treated ants.

## Additional Information and Declarations

### Data Availability

The following information was supplied regarding data availability: The raw data are provided in the Supplemental Files.
